# Race between virus and inflammasomes: inhibition or escape, intervention and therapy

**DOI:** 10.3389/fcimb.2023.1173505

**Published:** 2023-07-03

**Authors:** Nijin Wu, Chunzhi Zheng, Jiarui Xu, Shujun Ma, Huimin Jia, Meizhu Yan, Fuxiang An, Yi Zhou, Jianni Qi, Hongjun Bian

**Affiliations:** ^1^ Shandong Provincial Hospital Affiliated to Shandong First Medical University, Jinan, Shandong, China; ^2^ Shandong Provincial Hospital for Skin Diseases and Shandong Provincial Institute of Dermatology and Venereology, Shandong First Medical University & Shandong Academy of Medical Sciences, Jinan, Shandong, China

**Keywords:** inflammosome, viruses, innate immune, immune escape, therapeutic strategies

## Abstract

The inflammasome is a multiprotein complex that further regulates cell pyroptosis and inflammation by activating caspase-1. The assembly and activation of inflammasome are associated with a variety of diseases. Accumulative studies have shown that inflammasome is a key modulator of the host’s defense response to viral infection. Indeed, it has been established that activation of inflammasome occurs during viral infection. At the same time, the host has evolved a variety of corresponding mechanisms to inhibit unnecessary inflammasome activation. Therefore, here, we review and summarize the latest research progress on the interaction between inflammosomes and viruses, highlight the assembly and activation of inflammosome in related cells after viral infection, as well as the corresponding molecular regulatory mechanisms, and elucidate the effects of this activation on virus immune escape and host innate and adaptive immune defenses. Finally, we also discuss the potential therapeutic strategies to prevent and/or ameliorate viral infection-related diseases via targeting inflammasomes and its products.

## 1 Introduction

A wide range of pathogenic viruses, such as influenza viruses, severe acute respiratory syndrome coronavirus 2 (SARS-CoV-2), hepatitis B virus (HBV) and human immunodeficiency virus (HIV) type 1 (HIV-1), can cause severe diseases and threaten human health (Thomas et al., 2009; Ding et al., 2019; Jin et al., 2022; Junqueira et al., 2022). The innate immune system is a protective response to danger signals and is the first line of defense against invasive viral infections. During infection, pattern recognition receptors (PRRs) can recognize pathogen-associated molecular patterns (PAMPs) and damage-associated molecular patterns (DAMPs), which subsequently triggers host innate immune responses and inflammatory responses (Akira et al., 2006; Wang L. et al., 2021). The PRR described so far can be divided into toll-like receptors (TLRs), nucleotide-binding oligomerization domain (NOD)-like receptors (NLRs), cyclic GMP-AMP synthase (cGAS) - stimulator of interferon genes (STING), retinoic acid-induced gene (RIG)-I-like receptors (RLRs), C-type lectin receptors (CLRs) and absent in melanoma 2 (AIM2)-like receptor (ALRs) (Schroder and Tschopp, 2010). Thereinto, NLRs and ALRs are also important components of the inflammasome (Kanneganti, 2010).

The inflammasome is a multiprotein complex composed of sensors, adaptors and effector molecules (Kagan et al., 2014). In response to a variety of signals, it induces self-oligomerization and triggers the release of inflammatory cytokines and cell pyroptosis (Rathinam and Fitzgerald, 2016). According to the receptor proteins on the sensors, inflammasomes can be divided into NLRs and ALRs families. Common receptors for inflammasome in the NLRs family include NLRP1, NLRP3, NLRP6, NLRP7, NLRP12, neuronal apoptosis inhibitory protein (NAIP) and NLRC4, while that in the ALRs (also known as PYHIN) family include AIM2 and interferon-inducible protein 16 (IFI16) (Dadmanesh et al., 2019). Inflammasomes, like other innate immune-related receptor molecules, are involved in innate immune and inflammatory responses of pathogens in a wide array of viral infectious diseases. Upon infection, NLRs/ALRs recognize intracellular PAMPs and DAMPs, leading to the assembly of inflammasome and the activation of caspase-1. The process will turn pro-IL-1β and pro-IL-18 of non-biological activity into IL-1β and IL-18, which participate in innate immune defense and inflammation (Martinon et al., 2002). In addition, active caspase-1 also regulates an inflammatory programmed cell death called pyroptosis by cleaving gasdermin D (GSDMD) (Broz and Dixit, 2016).

Interestingly, recent studies have pointed that the activation of inflammosomes plays a key role in viral infection-related diseases. For instance, taking NLRP3 as an example, it has attracted much attention and is also the most extensively studied. Studies have shown that it is associated with the severity of SARS-CoV-2 infection and causes cardiac complications (Vora et al., 2021; Zhao N. et al., 2021). Furthermore, NLRP3 inflammosome enhances hepatitis C virus (HCV) replication (McRae et al., 2016) and promotes the expression of IL-1β and IL-18 in patients with HBV (Molyvdas et al., 2018). On the other hand, however, it is not surprising that viruses have also evolved different mechanisms to suppress inflammasome activation and thereby evade host immunity. In short, inflammasome plays a very vital role in many viral infections.

In response to the above mentioned, here, we summarize the different types of inflammasome involved in viral infection and their activation signals, focusing on the central role of inflammasome in the course of viral infection, and how viruses use inflammasome to escape the host immune response. Finally, based on a comprehensive understanding of the inflammasome function in viral infections, we also discuss the current advances in therapies and interventions targeting inflammasome in related viral infections.

## 2 Inflammasome

The canonical inflammasome complex consists of a cytosolic sensor (a member of the NLRs or ALRs protein), an adaptor (apoptosis-associated speck-like protein (ASC)), and an effector molecule (pro-caspase-1). ASC adaptor protein allows ALRs/NLRs to interact with pro-caspase-1 to form caspase-1, inflammasome assembly and priming of downstream reactions/responses such as IL-1β and IL-18 cytokines secretion and pyroptosis (Broz, 2019). Besides, there is a non-canonical inflammasome pathway targeting caspase-11 (in mice), caspase-4, and/or caspase-5 (in humans) (Downs et al., 2020). Previously, it was thought that RNA viruses activated NLRs inflammasome and DNA viruses activated ALRs inflammasome in the traditional view. However, in a recent review, it has pointed out that some viruses do not fit this classification, such as DNA virus herpes simplex virus 1 (HSV-1) inflammasome activation in macrophages that depends on NLRP3 but not AIM2 or IFI16, and NLRP3 inflammasome activation in THP1 cells infected with the RNA virus enterovirus 71 (EV71) (Wallace and Russell, 2022).Next, we will briefly introduce the structure and activation mechanism of different inflammasomes in this section.

### 2.1 NLRs

**Figure 1:**
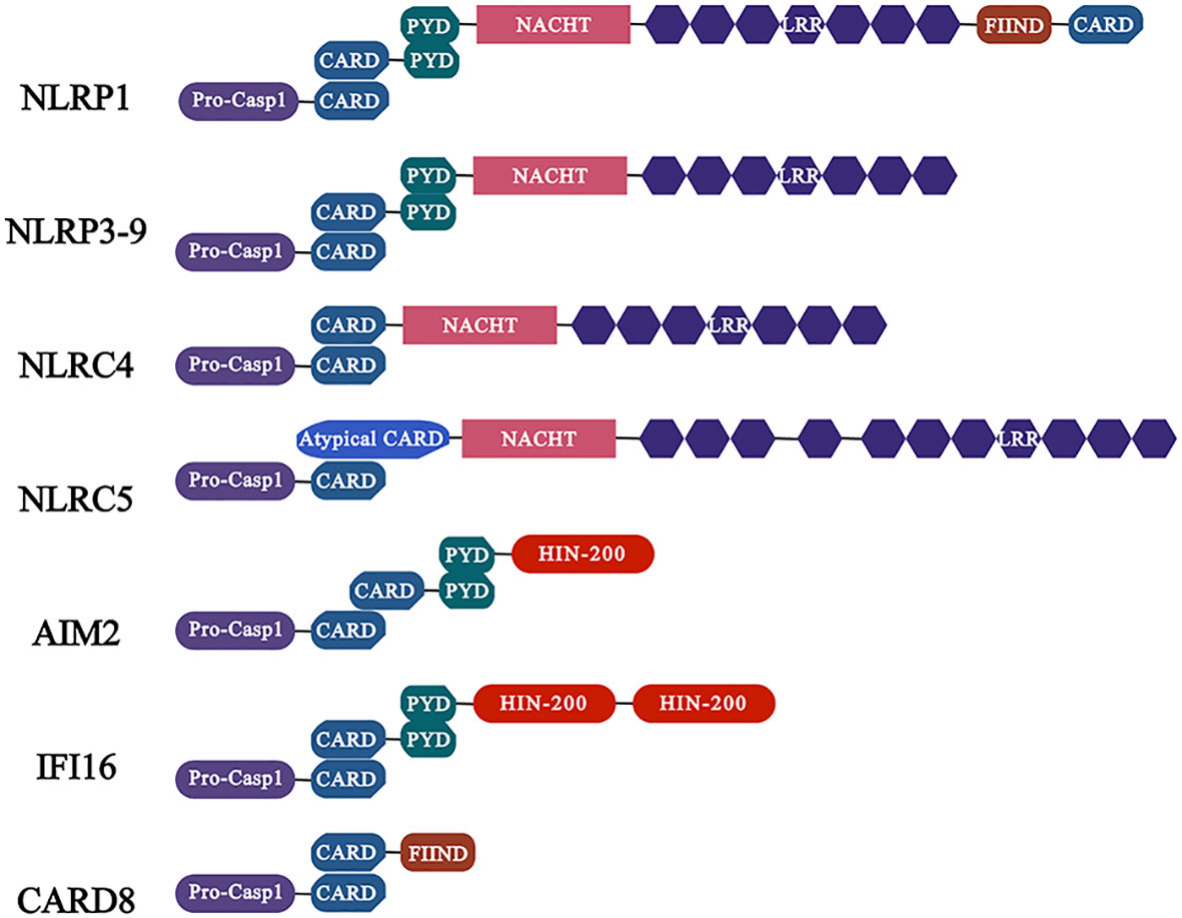
Characteristics of common inflammasomes and their domains. Members of the NLR family of receptors including NLRP1, NLRP3 and NLRC4 as well as ALR family members AIM2 and IFI16. Among them, the members of NLRP subfamily are characterized by the presence of PYD domain, while the NLRC subfamily members contain CARD, which activate caspase-1 (activation in NLRC4 is independent of ASC) through PYD-PYD and CARD-CARD interactions with ASC, and further regulate the release of pro-inflammatory cytokines.

NLRs are the best described in the family of inflammasome sensor protein. In summary, NLRs contains three domains: the variable N-terminal domain, which contains a pyrin domain (PYD) or a caspase activation and recruitment domain (CARD) that interacts with adaptor proteins or directly binds to enzymes such as pro-caspase-1 to recruit and activate the downstream signaling proteins; the central nucleotide domain (NOD, also known as the NACHT domain), which is essential for nucleotide binding and oligomerization; the C-terminal domain, which contains sequence of leucine-rich repeats (LRRs) motif to sense ligands and is considered as negative regulators of NLRs activation (Kanneganti, 2010). Furthermore, NLRs are divided into two families based on their N-terminal domains, namely NLRP with a PYD N-terminal domain and NLRC with a CARD N-terminal domain (de Carvalho Ribeiro and Szabo, 2022) (**Figure 1**).

#### 2.1.1 NLRP1

In, 2022, Martinon et al. identified the first inflammasome, NLRP1, in the monocyte cell line THP-1 (Martinon et al., 2002). Since then, the inflammasome NLRP1 has become the focus of research, and brought the inflammasome itself into people’s vision, making it one of the research hotspots pursued by researchers for last 20 years and thereafter. The NLRP1 protein contains five distinct domains: the amino-terminal PYD, the NACHT domain, six LRR domains, a function-to-find domain (FIIND) and a carboxy-terminal CARD (Mitchell et al., 2019). At present, how the NLRP1 inflammasome is activated by different pathogens is still not fully clarified, although there is evidence that the cleavage of NLRP1 is indispensable for its activation. Previously, Chavarría-Smith et al. discovered through experiments in rats and mice that the lethal factor (LF) of Bacillus anthracis lethal toxin (LeTx) is able to activate inflammasome by cleaving NLRP1B. LeTx is a two-component toxin, composed of the LF protease and the channel-forming protective antigen (PA) protein that transports LF into cells (Chavarría-Smith and Vance, 2013). Subsequently, it has been shown that N-terminal cleavage can significantly destabilize NLRP1B, leading to its ubiquitination and proteasome-mediated degradation *in vitro* (Sandstrom et al., 2019). This mechanism is associated with the special domain FIIND of NLRP1. More specifically, the FIIND, containing two sub-structural domains, ZU5 and UPA, auto-processes to produce two non-covalently linked polypeptides, nucleotide-binding domain (NBD)-LRR-ZU5 and UPA-CARD. The N-terminal domain of NLRP1 is degraded by proteasomes as a result of N-terminal cleavage, which releases the biologically active C-terminal UPA-CARD fragment. UPA-CARD self-assembles and forms a platform for pro-caspase-1 recruitment and activation (Sandstrom et al., 2019; Huang et al., 2021). In addition, it was recently found that the dipeptidyl peptidases (DPP) 8 and DPP9 interact with FIIND of NLRP1 and inhibit its spontaneous activation (Huang et al., 2021). DPP8 and DPP9 are related intracellular prolyl peptidases, acting as an endogenous inhibitor of human NLRP1 inflammasome (Chui et al., 2019; Huang et al., 2021). Moreover, the ZU5 domain is important for inhibiting DPP9 non-dependent UPA-CARD activation (Huang et al., 2021). In conclusion, N-terminal degradation of NLRP1 is the unifying mechanism of NLRP1 activation. As far, it has been proved that the 3C protease of small ribonucleic acid viruses (such as human rhinovirus, coxsackievirus B3) (Robinson et al., 2020), the open reading frame (ORF) 45 protein of Kaposi sarcoma-associated herpesvirus (KSHV) can activate NLRP1 (Yang et al., 2022), and the bovine pox virus F1L protein can mediate immune escape by inhibiting NLRP1 (Gerlic et al., 2013).

#### 2.1.2 NLRP3

NLRP3 is the most extensively studied inflammasome in various viral infections, and is also a major focus of the follow-up discussion in this review. We will introduce its role in different viral infections in detail in the following sections of viruses and inflammasome. NLRP3 protein consists of three domains of the NLR family described above. In response to different danger signals, NLRP3 lead to disassembly of the transGolgi network (TGN), and NLRP3 is recruited to the dispersed TGN (dTGN) via phosphatidylinositol-4-phosphate (PtdIns4P). Then dTGN serves as a scaffold for NLRP3 aggregation into multiple puncta, which resulted in ASC polymerization (Chen and Chen, 2018). Subsequently, ASC recruits pro-caspase-1 through CARD which resulted in activation of NLRP3 and signaling cascade. In general, regulation of NLRP3 inflammasome occurs in two steps: priming and activation. In the priming step, PAMP or DAMP is recognized by TLR, which upregulates NLRP3 and pro-IL-1β expression through myeloid differentiation factor 88 (MYD88) and TIR-domain-containing adaptor protein inducing IFN-β (TRIF) dependent signaling pathways. In addition to promoting the transcription of inflammasome components, priming step also comprises post-translational modifications of NLRP3, including ubiquitination, phosphorylation and sumoylation, to stabilize it in an autoinhibited inactive state thereby rapidly responding to the stimulus (Swanson et al., 2019). Notably, before activation it must be primed. In the activation step, NLRP3 oligomer, ASC and pro-caspase-1 are assembled into the oligomerization to form a complex (Kelley et al., 2019). Cellular signaling events causing this step include calcium ion (Ca^2+^) influx, potassium ion (K^+^) efflux, reactive oxygen species (ROS) production, mitochondrial dysfunction, lysosomal rupture and intracellular chloride ion channel-dependent Cl^-^ efflux. Among them, K^+^ efflux was shown to be the most important activator (de Carvalho Ribeiro and Szabo, 2022). In addition, several crucial regulators of NLRP3 inflammasome activation have also been reported. For example, double-stranded RNA-dependent protein kinase (PKR) interacts with NLRP3 inflammasome to stimulate the secretion of downstream IL-1β, IL-18 and high mobility group box 1 (HMGB-1) both in human THP1 cell and mice bone marrow-derived macrophages (BMDMs) (Lu et al., 2012). Guanylate-binding protein 5 selectively facilitates NLRP3 inflammasome assemble with pathogenic bacteria or adenosine triphosphate stimulated *in vivo* cell based assay (Shenoy et al., 2012). Accumulative studies have confirmed that NLRP3 inflammasome are involved in the pathogenesis of several viral infections, and these viruses may also be able to target inflammasome to mediate their immune escape.

#### 2.1.3 NLRP6

NLRP6 (originally known as PYPAF5) is a novel NLR family member. Its signaling regulatory mechanisms, structural assembly, and the role in host defense have not been elucidated yet to date. NLRP6 protein is primarily expressed in lung and liver, but the highest expression is in intestine. As other NLR family members, NLRP6 consists of an N-terminal PYD, a central NACHT domain, and a C-terminal LRR domain (Zheng D. et al., 2021). Recently, Shen et al. found that when interact with ligands, an intrinsically disordered poly-lysine sequence (K350-354) of NLRP6 mediate the formation of liquid-liquid phase separation (LLPS), which is important for inflammasome activation (Shen et al., 2021). Like NLRP3, the activation of NLRP6 requires two steps: priming and activation. Among them, microbial signals like type I IFN (IFN-I) and metabolic signals like peroxisome proliferator-activated receptor-γ (PPAR-γ) activator may act as priming signals for activation of the NLRP6 inflammasome (Li and Zhu, 2020). It was displayed that NLRP6 expression is regulated by IFN-I and type III IFN (IFN-III) via IRF3/7, suggesting that NLRP may belong to IFN-stimulated genes (ISG) (Wang P. et al., 2015). Also, NLRP6 is able to bind viral RNA via the RNA helicase Dhx15 and interact with mitochondrial antiviral signaling protein (MAVS) to induce the expression of IFN-I, IFN-III and ISG (Wang P. et al., 2015). In addition, Kempster et al. found that transcription factor binding analysis of the rat array data shown the promoter region of NLRP6 contains binding sites for PPAR-γ, retinoid X receptor-α (RXR-α) and chicken ovalbumin upstream promoter transcription factor 1 (COUP-TF1). By treating human intestinal epithelial (Caco-2) cells with rosiglitazone, a PPAR-γ agonist, the expression of NLRP6 mRNA was increased more than 2-fold (Kempster et al., 2011). Microbial components, such as metabolites, virus RNA, bacterial lipoteichoic acid, and lipopolysaccharide (LPS) may directly bind to NLRP6 and act as secondary signals to induce the assembly of inflammasome (Li and Zhu, 2020). PYD of NLRP6 promotes ASC-PYD polymerization to further activate caspase-1 as well as caspase-11 (Li and Zhu, 2020).

#### 2.1.4 NLRC4 (IPAF)

NLRC4 (also known as IPAF) is expressed in myeloid cells (Sutterwala and Flavell, 2009), Unlike other NLRs inflammasome family member, it is not a direct sensor of NLRC inflammasome ligands. The adaptor protein NLRC4 and the NLR family of NAIP collaborate to form the inflammasome (Lee et al., 2018). Structurally, NAIP and NLRC4 proteins share two domains. One domain is NACHT domain, which is comprised of a NBD, Helical Domain 1 (HD1), Winged Helix Domain (WHD) and HD2, and another one domain is LRR. In addition, there are three Baculovirus inhibitor-of-Apoptosis repeat (BIR) domains in NAIP, while NLRC4 contains a CARD domain (Bauer and Rauch, 2020). Type III secretory apparatus (Miao et al., 2010) as well as bacterial flagellin (Amer et al., 2006) are ligands for activation of NAIP/NLRC4 inflammasome. The BIR domain of NAIP allows it to engage flagellin, which may be critical for inflammasome formation (Tenthorey et al., 2014). Besides, cryo–electron microscopy (cryo-EM) structural analysis has shown that in addition to the BIR domains, the N-terminal helix, HD1, HD2, an insertion domain (ID), and the LRRs all contribute to flagellin binding (Tenthorey et al., 2007). Without the help of the adaptor protein ASC, the CARD domain of NLRC4 is able to bind with the CARD domain of caspase-1 directly. Nevertheless, when NLRC4 bind with the ASC, it leads to more efficient caspase-1 activation (Broz et al., 2010). Furthermore, NLRC4 was able to recruit caspase-8 after binding ASC (Lee et al., 2018). In NAIP and NLRC4, the LRR domain was indicated to be essential for self-inhibition. Self-inhibition is promoted by LRR-NBD contact directly, which is further stabilized by ADP binding to the NBD (Hu et al., 2013). After ligand binding with NAIP, this kind of self-inhibition is relieved. Recently, it was discovered that the first non-bacterial ligand, HIV, is also capable of activating NLRC4, the exact mechanism of which will be described in the section of HIV and inflammasome.

### 2.2 ALRs

In addition to NLRs, ALRs are also sensor proteins that play a key role in inflammsome (Fernandes-Alnemri et al., 2009). ALRs contain HIN and PYD domains. The non-specific DNA recognition by members of the ALRs family is achieved by electrostatic attraction between the positively charged residues in HIN domain and the glycophosphate backbone of double-stranded DNA (dsDNA), resulting in the release of intramolecular complexes of PYD and HIN domains in an autoinhibited state, and facilitating the assembly of inflammasome (Jin et al., 2012) (**Figure 1**). Notably, AIM2 senses viral DNA in cytoplasm, whereas IFI16 detects viral DNA in cytoplasm and nucleus.

#### 2.2.1 AIM2

The AIM2 protein, which is consist of a C-terminal HIN domain and an N-terminal PYD domain, is a member of the IFN-inducible p200-protein family (Veeranki and Choubey, 2012). AIM2 is highly expressed in lymph nodes, appendix and spleen. It recognizes dsDNA through the HIN domain and interacts with the N-terminal PYD. The PYD-connected ASC induces caspase-1 recruitment through the CARD of ASC and form the AIM2 inflammasome. However, it is noteworthy that the DNA recognized by AIM2 must meet the length requirement of more than 80bp, but there is no requirement for DNA sequence, GC content or origin (Wang and Yin, 2017). AIM2 inflammasome is able to induce the maturation and the release of inflammatory cytokine such as IL-1β and IL-18, triggering an inflammatory response (Wang et al., 2019). Several factors are known to regulate the expression of AIM2. Firstly, IFN-γ promotes the expression of AIM2. Besides, epigallocatechin gallate (EGCG), EFLA-945, obovatol, withaferin and RGFP966 have inhibitory effects on AIM2 (Wang et al., 2022). A number of DNA viruses, such as human cytomegalovirus (Huang et al., 2017), human papilloma virus (Reinholz et al., 2013), HBV (Pan et al., 2016), are known to activate AIM2 inflammasome. However, AIM2 does not identify all DNA viruses, such as HSV-1, which maybe because viral infection inhibits DNA-AIM2 interactions (Maruzuru et al., 2018).

#### 2.2.2 IFI16

IFI16 inflammasome, another ALRs family member, also plays an indispensable role in the host defense against viruses. Similar to AIM2 described above, IFI16 contains an N-terminal PYD and two C-terminal HIN200 domains (AIM2 contains a HIN200) (Caneparo et al., 2018). These two HIN domains may play opposite roles in the IFI16 inducing the production of IFN-β. It has been demonstrated that absence of HINb impairs IFN-β induction by IFI16, while impaired HINa is able to inversely enhance its ability to mediate IFN-β production (Ni et al., 2016). Under physiological conditions, the expression of the IFI16 is restricted to vascular endothelial cells, keratinocyte cells and hematopoietic cells (Caneparo et al., 2018). Particularly, so far, IFI16 is the only receptor that senses DNA in the cytoplasm and nucleus to form inflammasome (Unterholzner et al., 2010). In unstimulated cells, IFI16 is predominantly expressed in the nucleus. IFI16 can sense and bind viral DNA through its HIN domain and recruit ASC to initiate caspase-1 activation, assembling to form inflammasome and also stimulate IFN-I expression through the STING-IRF axis (Jakobsen and Paludan, 2014; Caneparo et al., 2018). IFI16, as a key DNA sensor, has now been verified to be involved in defense against viruses by inducing inflammasome assembly in epstein-Barr Virus (EBV) (Ansari et al., 2013), and HSV (Johnson et al., 2013). However, vivo based assay shown that Human papillomavirus (HPV) is capable of mediating the degradation of IFI16 inflammasome by the ubiquitination/proteasome pathway through the interaction of Tripartite motif-containing protein 21 (TRIM 21) with IFI16, thereby inhibiting pyroptosis mediated immune escape (Song et al., 2020).

## 3 Severe acute respiratory syndrome coronavirus 2 and inflammasome

In December, 2019, a case of aggregated pneumonia of unknown cause broke out in Wuhan, China. The disease was subsequently named Coronavirus Disease, 2019 (COVID-19), and its pathogen was SARS-CoV-2 (Merad et al., 2022). As of February, 2023, there are over 757 million confirmed cases worldwide, resulting in 6.85 million deaths (WHcoronavirus (COVID-19) dashboard). The majority of patients with COVID-19 are asymptomatic/mildly infected, but a small proportion of patients develop severe complications, resulting in systemic inflammation, tissue damage, acute respiratory distress syndrome, thromboembolic complications, heart damage and/or cytokines storm (Diamond and Kanneganti, 2022). Currently, several studies have demonstrated the involvement of inflammasome in the pathogenesis of COVID-19 (**Table 1**). The activation of inflammasome is likely to be involved in the formation of severe cytokine storms, leading to ARDS, MODS, and even ultimately death (Kaivola et al., 2021). On the one hand, Baena Carstens et al. reported that biomarkers (ACE2, NF-κB, NOX4, ASC), pyroptosis and inflammasome-derived cytokines such as IL-18 and caspase-1 were all significantly increased in the COVID-19 group via immunohistochemistry of lung tissue from patients who died of SARS-CoV-2 infection (Baena Carstens et al., 2022). Meanwhile, a recent study found that in monocytes from COVID-19 patients, ASC co-localized with NLRP3 and AIM2, but not pyrin, meanwhile most zombie cells had ASC specks (62 ± 9%). This suggests that the vast majority of monocyte death is associated with pyroptosis caused by AIM2 and NLRP3 inflammasomes (Junqueira et al., 2022). On the other hand, inflammasome inhibitors can effectively inhibit the activation of inflammasome in PBMC of COVID-19 patients and SARS-CoV-2 infected mouse models, so as to suppress the production of its downstream cytokines (de Almeida et al., 2022). The IL-1 receptor blocker Anakinra has been clinically displayed to saliently reduce COVID-19-related mortality (Huet et al., 2020). These results about the histopathology of patients with SARS-CoV-2 infection and the treatment of animal models above all imply that inflammasome really play an important role in the pathogenesis of COVID-19. However, the specific role of SARS-CoV-2 and its related proteins in the activation and assembly of inflammasome needs to be further revealed.

**Table 1:** The Role played by different inflammasomes in various viruses.

Types of viruses	Expression	Types of inflammasome	Influence	References
SARS-CoV-2	Monocytes	NLRP3	Inflammatory cell death that aborts the production of infectious virus but causes systemic inflammation	(Junqueira et al., 2022)
	Monocytes	AIM2	Inflammatory cell death that aborts the production of infectious virus but causes systemic inflammation	(Junqueira et al., 2022)
	Pulmonary tissue	NLRP3	Increasing expression of IL-1β and IL-18, which are some of the cellular products of acute inflammasome activation	(Baena Carstens et al., 2022)
	A549 cell line	NLRP3	Activating the inflammasome to cleave pro-caspase 1, pro-IL-1β, and the pore-forming Gasdermin D, inducing cell death	(Xu et al., 2022)
	Human bronchial and lung epithelial cell lines	NLRP3	Pyrotosis of lung epithelial cells	(Sun et al., 2022)
	Lung epithelial cells	NLRP1	Limitation of the production of infectious viral particles	(Planes et al., 2022)
IAV	BMDCs, DMDMs, normal human bronchial epithelial	NLRP3	Induction of apoptosis, necroptosis, and pyroptosis in IAV-infected cells	(Kuriakose et al., 2016)
	and alveolar macrophages	AIM2	IAV-stimulated proinflammatory response	(Zhang et al., 2017)
HIV	monocytes	NLRP3	Exacerbating inflammation in a mouse model of footpad swelling	(Rossol et al., 2012)
	microglia	NLRP3	Causes brain disease and neuroinflammation	(Walsh et al., 2014; He et al., 2020)
	CD4+ central and effector memory T cells	IFI16	Promotes CD4^+^ T cell pyroptosis	(Nissen et al., 2014; Kearns et al., 2018)
	macrophages	IFI16	Restrict HIV-1 replication	(Hotter et al., 2019)
	peripheral blood	PHYIN protein family	Promote structural and functional deterioration of GALT in patients	(Feria et al., 2018)
	GALT and PBMC	NLRP1	Enhanced HIV-1 replication	(Feria et al., 2018)
	dendritic cell	NLRC4	Promotes dendritic cell activation in HIV-1 patients	(Dos Reis et al., 2019)
	microglia	NLRC5	Results in increased expression of proinflammatory cytokines	(Periyasamy et al., 2019)
	CD4+ T cell and macrophages	CARD8	Induced pyroptosis in HIV-1-infected cells	(Wang Q. et al., 2021)
HBV	liver tissues	NLRP3	Positively correlated with HBV DNA concentration	(Xie et al., 2020)
	PBMC	AIM2	Involved in HBV immune clearance	(Wu et al., 2013)
	hepatocyte	AIM2	Promote liver inflammation in CHB patients	(Han et al., 2015)
	PBMC	IFI16	Promotes HBV clearance	(Wieland et al., 2004; Yang et al., 2020)
Rotavirus	intestinal epithelial cells	NLRP9B	Promotes clearance of the virus	(Zhu et al., 2017)
ECMV	mouse intestinal epithelial cells	NLRP6	Control of enterovirus infections	(Wang P. et al., 2015)
WNV	neurons	NLRP3	Inhibition of virus replication in neurons	(Ramos et al., 2012)
HCV	RAW 264.7	NLRP3	Triggers liver inflammation	(Farag et al., 2017)

**Figure 2:**
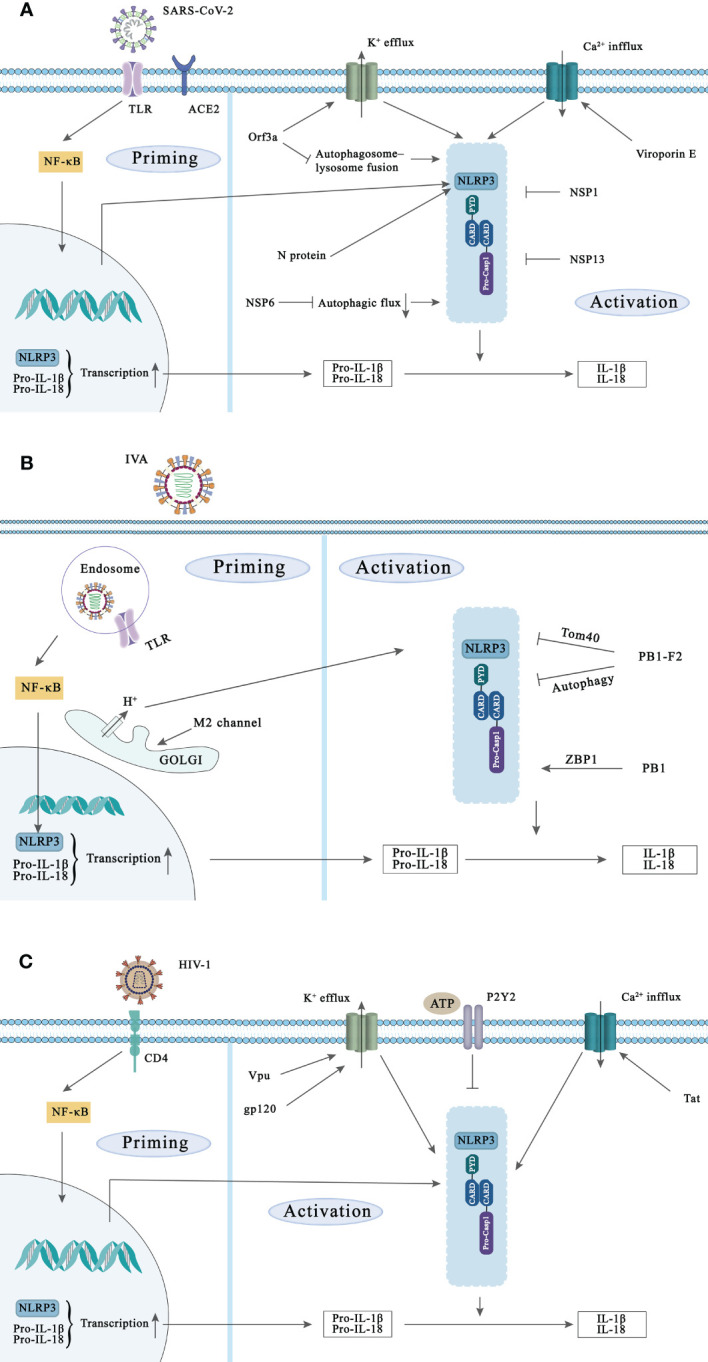
NLRP3 inflammasome activation during SARS-CoV-2, IVA, and HIV-1 infection and the strategies of these viruses to evade NLRP3 inflammasoma-mediated innate immunity. Activation of the NLRP3 inflammasome requires two signals. Priming signal: Viral components are recognized by PRRs such as TLR and NLR and induce NF-κB activation, leading to the transcription and expression of NLRP3, pro-IL-1β, and pro-IL-18. Subsequently, NLRP3 is activated in response to multiple DAMPs and PAMPs induction such as lysosomal damage and abnormal ion flux, leading to caspase-1 activation. **(A)** SARS-CoV-2 initiates NF-κB-mediated transcription and translation of NLRP3 gene by activating TLR complement receptor and ACE2. Viroporins E and ORF3a proteins can promote the activation of NLRP3 inflammasome through ion flux. In addition, N protein and NSP6 can also activate NLRP3 inflammasome, while NSP1 and NSP13 of SARS-CoV-2 can inhibit NLRP3 inflammasome activation. **(B)** Recognition of IVA by TLR signaling induces transcription of NLRP3. The ion channel imbalance of M2 in the Golgi and PB1 can activate the NLRP3 inflammasome. Conversely, PB1-F2 can also inhibit the inflammasome activation of NLRP3 in multiple ways. **(C)** HIV-1 infects target cells via the CD4 receptor, and various components of HIV-1 can increase the transcription of NLRP3, pro-IL-1β and pro-IL-18. In addition, Vpu, Tat and gp120 in Play a role in the activation of NLPR3 inflammasome. The activated P2Y2 facilitates HIV-1 virus infection by degrading NLRP3.

SARS-CoV-2 is an enveloped, positive-sense, single-stranded RNA virus of the β-coronavirus family, which belongs to the same category as SARS-CoV, the pathogen of the, 2002-2004 SARS epidemic, and MERS-CoV, the pathogen of Middle East Respiratory Syndrome (Jackson et al., 2022). The structural proteins, the non-structural proteins (NSPs), and the accessory proteins are three basic categories of viral proteins encoded by SARS-CoV-2. These three protein families are in charge of a certain task during the viral life cycle. Structural proteins contain the spike (S), envelope (E), membrane (M), and nucleocapsid (N) proteins, and accessory proteins include ORF 3a, ORF3b, ORF3c, ORF3a, ORF7b, ORF8, ORF9b, ORF9c and ORF10. More detailed researches have pointed out that SARS-CoV-2 related proteins directly and/or indirectly promote the activation and assembly of inflammasome through multiple mechanisms (**Figure 2**). For example, after SARS-CoV-2 enters our body, its channel proteins oligomerization forms pores, invades the host cells, and then affects the physiological homeostasis of these cells, which is one of the key factors of viral pathogenicity during viral infection  (Zhao N. et al., 2021). Among 29 proteins of SARS-CoV-2, the E and ORF3a proteins have been identified as viroporins. Viral channel proteins may activate the NLRP3 inflammasome as well as contribute to the massive release of inflammatory cytokines. The E protein of SARS-CoV-2 is the ligand of TLR2, one of the primary members of PRR family, and their binding causes the priming of NLRP3 inflammasome (Zheng M. et al., 2021). Another viral channel protein ORF3a also plays a significant role in promoting the assembly of inflammasome. ORF3a protein activates NLRP3 inflammasome by causing K^+^ efflux, and then triggers interactions of NIMA-related kinase 7 (NEK7) and NLRP3, leading to ASC and caspase-1 recruitment (Xu et al., 2022). Besides, existing data has verified that ORF3a of SARS-CoV could promote TRAF3-dependent ubiquitination of ASC to activate NLRP3 inflammasome (Siu et al., 2019). Recently, a team found that ORF3a co-localizes with lysosomes and interacts with VPS39 to inhibit autophagic flux (Zhang Y. et al., 2021), which is another pathway for ORF3a to activate NLRP3 inflammasome. Moreover, N protein interacts directly with the NLRP3 protein to promote the binding of it and ASC and further accelerate NLRP3 inflammasome assembly by ex vivo and *in vivo* assay (Pan et al., 2021). According to a recent study, NSP6, the NSPs of SARS-CoV-2, interacts with ATP6AP1 (a vacuolar ATPase proton pump component) in lung epithelial cells, leading to the stagnation of autophagic flux and thereby producing an inhibitory effect on lysosomal acidification (Sun et al., 2022). Namely it indirectly inhibits inflammasome activation via autophagy. In addition to NLRP3, which is the most studied at present, it has been shown that NLRP1 is cleaved at the Q333 site by the multiple coronavirus 3CL protease NSP5, thereby triggering inflammasome assembly and cell death to limit the production of infectious viral particles (Planes et al., 2022).

Interestingly, the targeting of the inflammasome or pyroptosis is one of the many ways that viruses can mediate immunological escape to evade host immune surveillance. As reported by Yalcinkaya M et al., the E protein of SARS-CoV-2 may initially inhibit the response of host NLRP3 inflammasome to viral RNA, but may increase the response of NLRP3 inflammasome in the later stage of infection both in human macrophages and mice model (Yalcinkaya et al., 2021). The exact mechanism of this phenomenon is not known. However, there is no doubt that the virus suppresses the host’s immune response at the early stage of replication, and undergoes activation of inflammasome at the late stage of replication, both of which are harmful to our host. In addition, in SARS-CoV-2 infected monocytes, IL-1β expression was enhanced, but its secretion was reduced. This strategy may help the virus to evade immune attack during the early stages of infection (Ma et al., 2021). Furthermore, two NSPs of SARD-CoV-2, NSP1 and NSP13, inhibit caspase-1 mediated IL-1β secretion, indicating they are potent antagonists of NLRP3 inflammasome in THP1 cells (Kim et al., 2021). The N protein of SARS-CoV-2 binds to the GSDMD junction region, and blocks the processing of GSDMD by caspase-1, thereby resisting pyroptosis (Ma et al., 2021).

## 4 Influenza viruses and inflammasome

Influenza viruses are enveloped RNA viruses of the Orthomyxoviridae family, including four different virus subtypes: A, B, C and D (Ong et al., 2017; Long et al., 2019). Influenza A viruses (IAV) generally infects a variety of host species, influenza B and C viruses infecthumans, and influenza D viruses mainly infect cattle, goats and pigs (Long et al., 2019). Influenza viruses are one of the most common causes of respiratory infection in humans, resulting in high morbidity and mortality. Since, 1700, there have been approximately a dozen pandemics of IAV. The most notable is the H1N1 “Spanish” influenza pandemic in, 1918, which is estimated to cause 50 to 100 million people death (Ong et al., 2017). Influenza B viruses can periodically cause large-scale epidemics, but it will not cause a pandemic. Influenza C viruses are endemic and occasionally cause mild respiratory illness (Taubenberger and Morens, 2008). IAVs are classified into different subtypes based on the viral surface proteins, hemagglutinin (HA) and neuraminic (NA). So far, 15 HA subtypes (H1-H15) and 9 neuraminidase subtypes (N1-N9) of IAVs have been identified (Nicholson et al., 2003).

Studies have indicated that the activation of inflammasome during IAV infection is a double-edged sword for the host. On the one hand, NLRP3 inflammasome plays a protective role during severe H1N1 infection. The caspase-1^-/-^ and NLRP3^-/-^ mice have shown faster death from H1N1 infection than the wild type mice (Thomas et al., 2009). On the other hand, cytokine storm caused by highly pathogenic IAV infection is thought to be a critical factor for high mortality (**Figure 2**). Mice that the NLRP3 inflammasome component knocked out, including NLRP3^-/-^, caspase-1^-/-^ and ASC^-/-^ were less susceptible to H7N9 virus attack than wild controls (Ren et al., 2017). Meanwhile, Tate et al. found that blocking NLRP3 at different stages of fatal IAV infection may be protective or detrimental. One day after IAV attack mice, they administrated the specific NLRP3 inhibitor MCC950 to these mice, resulting in high mortality. In contrast, administration of MCC950 at the peak of disease (a more likely clinical scenario) protects mice from severe IAV-induced disease (Tate et al., 2016). In the case of IAV infection, the sensors that activate the inflammasome include TLR3, TLR7 and RIG-1. TLR3 is activated by dsRNA produced during IAV infection, while TLR7 interacts with ssRNA in the endosomal membrane. RIG-I recognizes cytoplasmic ssRNA to cause inflammation and antiviral reaction through the transcription factors NF-κB and IRF interacting with MAVS Sarvestani and McAuley, (2017). Recently, Kuriakose et al. found that during IAV infection, IFN-inducible protein Z-DNA binding protein 1 (ZBP1), acting as a sensor for IAV nucleoprotein and polymerase subunit polymerase basic protein 1 (PB1), mediates the activation of NLRP3 inflammasome through the RIPK3-caspase-8 axis. Moreover, deletion of ZBP1 completely eliminated IAV-induced the activation of inflammasome, whereas deletion of RIG-I only partially affected the assembly of NLRP3 inflammasome. This may be related to the ability of RIG-I to induce the transcription and expression of IRF. They have also been demonstrated that ZBP1 is only necessary for the activation of NLRP3 inflammasome required for IAV infection, but is dispensable for other RNA viruses (e.g. vesicular stomatitis virus (VSV)) (Kuriakose et al., 2016).

In addition to promoting the activation of NLRP3 inflammsome through various ways, people found that IAV can also activate other receptor protein-mediated inflammasomes. For example, it has been uncovered that IAV infection enhanced AIM2 expression, the cleavage of caspase-1 and the release of IL-1β in the lung. Moreover, AIM2-deficient mice had reduced lung injury compared to the control group, which improved the survival rate of mice infected with IAV, but did not alter viral load (Zhang et al., 2017). Additionally, Lee et al. identified human myxovirus resistance protein 1 (MxA) as a functional inflammasome sensor in respiratory epithelial cells in, 2019. It recognized IAV nucleoprotein and triggered ASC spot formation through the interaction of its GTPase domain with the PYD domain of ASC (Lee et al., 2019).

The genome of IAV is composed of eight negative-stranded RNA fragments. The three largest RNA fragments encode three RNA polymerases, including polymerase acidic protein (PA), PB1 and PB2. The RNA fragment of PB1 also encodes a NSP, PB1-F2. Three medium-size RNA fragments encode HA, NA and nucleoprotein. The larger one of the remaining two fragments encodes the M1 matrix protein and the M2 ion-channel protein, and the smaller one encodes two NSPs, NS1 and NS2 (Das et al., 2010). Viral proteins are involved in the activation and inhibition of inflammasome (**Table 1**). Firstly, the M2 protein is an ion-selective channel protein that is required for the activation of inflammasome. M2-induced inflammasome activation is localized to the Golgi apparatus and is dependent on the pH gradient. Precisely, the ion channel activity of M2 allows the export of H^+^ from the acidified Golgi, which triggers the inflammasome activation (Ichinohe et al., 2010). The transmembrane domain of the M2 protein forms the channel pore in which His37 and Trp41 are located. His37 is the ion selective sensor and Trp41 is the gated sensor (Pandey and Zhou, 2022). Furthermore, PB1-F2 is a NSP localized to IAV mitochondria and is known to be a virulence factor regulating the innate immune response and NLRP3 inflammasome activity. PB1-F2 also stabilizes the auto-repressed and closed state of NLRP3 through interaction with the LRR and PYD of NLRP3 and prevents NLRP3 from binding to NEK7 (Boal-Carvalho et al., 2020). It is known that NEK7 is a host kinase that only binds to the open/active conformation of NLRP3. Moreover, PB1-F2 protein of IAV acts as an autophagy receptor through its LIR motif interacting with TUFM and LC3B, that mediated the autophagosome formation, to induce mitochondrial autophagy, thereby further inhibiting inflammasome activation (Wang R. et al., 2021). PB1-F2 fully translocates to the inner mitochondrial membrane through Tom40 channels and accelerates mitochondrial fragmentation by reducing the inner mitochondrial membrane potential, further inhibiting the RIG-I signaling pathway and the activation of NLRP3 inflammasome (Yoshizumi et al., 2014). Further, NS1, another NSP, enhanced MLKL-mediated NLRP3 inflammasome activation by interacting with MLKL, leading to increased synthesis and secretion of IL-1β (Gaba et al., 2019). TRIM25 is a positive regulator of porcine NLRP3 inflammasome-mediated IL-1β production. However, the NS1 C-terminus of pandemic IAV in, 2009 inhibited the interaction between caspase-1 and ASC by interacting withTRIM25. This reflects the inhibition of NS1 on the host-promoted inflammasome assembly process (Park et al., 2021). Also, the viral polymerase PB2 (residue E627K) has been shown to be a virulence factor for H9N2 avian influenza viruses in mice. NLRP3, IL-1β and TNFα levels were increased more in mice during infection when the virus carried the virulence marker of K627 compared to that of E627 (Yu et al., 2014).

## 5 Human immunodeficiency virus type 1 and inflammasome

HIV-1 is a spherical retrovirus composed of core and envelope. The core of the virus contains viral RNA genome, core structural proteins and a variety of enzymes (such as reverse transcriptase (RT), integrase (IN), protease (PR)) required for viral replication (Engelman and Cherepanov, 2012). The HIV genome is composed of two identical positive RNA strands. Among them, the three largest reading frames encode three main structural proteins Gag, Pol and Env (Turner and Summers, 1999). In addition, the HIV genome also encodes regulatory proteins transactivator of transcription (Tat) and Rev as well as accessory proteins Nef, Vif, Vpu and Vpr. The core is covered by an envelope embedded with glycoprotein gp120 and transmembrane protein gp41, both of which are encoded by the env gene (Huerfano et al., 2022). HIV-1 was first discovered in the summer of, 1983 (Worobey et al., 2016), which can make patients suffer from acquired immunodeficiency syndromes (AIDS) after infecting human body (Deeks et al., 2015). According to the WHO, more than 38.4 million people were infected with HIV in, 2021, of which nearly 2 million new cases occurred each year (Global Council on Inequality, AIDS and Pandemics).

As the name suggests, AIDS is characterized by severe immunodeficiency and persistent inflammation (Shi et al., 2022). HIV-1 mainly destroys the immune system by attacking host immune cells (mainly CD4^+^ T cells), so a marked reduction of circulating CD4^+^ T cells is a major feature of AIDS (Galloway et al., 2015). In fact, most of the dead CD4^+^ T cells are resting non-permissive cells in lymphoid tissue (Doitsh et al., 2014) that cannot complete the viral replication cycle, resulting in incomplete transcription of viral DNA and abortive (“bystander”) infections (Finkel et al., 1995; Galloway et al., 2015). Interestingly, the death of these “bystander” CD4^+^ T cells is mainly caused by pyroptosis mediated by inflammasome (Doitsh et al., 2010). To date, activation of multiple inflammasomes including NLRP3, NLRC4 and IFI16 has been reported in HIV-1 infection, while numerous studies have also found that HIV-1-induced inflammatory responses and pyroptosis are also dependent on inflammasome (**Table 1**) (Zhang C. et al., 2021).

NLRP3, as one of the most commonly studied inflammasome, has been identified in AIDS patients (Pontillo et al., 2012). Although there is no evidence that NLRP3 directly recognizes HIV-1, researchers have verified that various components of HIV-1 (e.g. gp120 and genomic RNA) are able to induce the activation of NLRP3 inflammasome in the last decade (**Figure 2**). Several studies indicated that HIV-1-derived RNA can trigger the first signal of NLRP3 activation in monocytes through TLR8 to promote NLRP3 transcription (Guo et al., 2014; Campbell et al., 2021), followed by ROS, Ca^2+^ influx, K^+^ efflux and many other endogenous agonists leading to activation of the NLRP3 inflammasome (Rossol et al., 2012; He et al., 2020; Stunnenberg et al., 2021). Currently, the regulatory proteins Tat and glycoprotein gp120 have been shown to induce NLRP3 inflammasome activation through ion flux. Differently, the regulatory protein Tat enhances Ca2^+^ influx (Rossol et al., 2012), while glycoprotein gp120 promotes K^+^ efflux (He et al., 2020). Interestingly, as a viroporins, Vpu not only triggers NLRP3 priming signals via TLR4, but also induces K^+^ efflux to trigger NLRP3 inflammasome activation (Triantafilou et al., 2021). And it has been reported that single nucleotide polymorphisms (SNPs) in the 3’-untranslated region (3’-UTR) of NLRP3 gene may be associated with susceptibility to HIV-1 infection (Pontillo et al., 2010).

However, with the deepening of research, people found that NLRP3 inflammasome activation has a dual function in HIV-1 infection. On the one hand, NLRP3 inflammasome enhance the body’s ability to clear the virus at the initial stage of HIV-1 infection as described by Reis et al. (2019). On the other hand, NLRP3 inflammasome promotes pyroptosis of “bystander” CD4^+^ T cells (Montague-Cardoso, 2021; Zhang C. et al., 2021) and causes brain disease and neuroinflammation that are closely associated with NLRP3 inflammasome activation in microglia (Walsh et al., 2014; He et al., 2020). Finally, surprisingly, Paoletti et al. demonstrated that HIV-1 induces NLRP3 degradation through purinergic receptor (P2Y2) signaling pathway, suggesting that HIV-1 has evolved an NLRP3-mediated immune escape strategy (Paoletti et al., 2019).

In addition, IFI16, as a sensor of viral DNA, belongs to the PYHIN protein family, and recognizes incomplete reverse transcript products of HIV-1, further enabling IFI16 inflamomsome assembly (Monroe et al., 2014). It has reported that IFI16 expression was significantly upregulated in HIV-1-infected patients (Nissen et al., 2014). This phenomenon has also been observed in rhesus macaques infected with simian immunodeficiency virus (SIV) (Kearns et al., 2018). In addition, both studies have indicated that IFI16 expression was positively correlated with high viral load and negatively correlated with the number of CD4^+^ T cells *in vivo*. This implies that IFI16 inflammosome promotes CD4^+^ T cell pyroptosis. However, in contrast, IFI16 has been verified to restrict HIV-1 replication in macrophages (Hotter et al., 2019). This hints that IFI16 plays different roles in different cells. And, AIM2, another member of PYHIN protein family, was also able to be activated by HIV-1 dsDNA (Burckstummer et al., 2009; Fernandes-Alnemri et al., 2009). It is worth noting that, in addition, Feria et al. pointed that the PHYIN protein family can also promote structural and functional deterioration of gut-associated lymphoid tissue (GALT) in HIV-1 infected patients (Feria et al., 2018). These evidences uncover that PYHIN inflammasome play a crucial role in innate immunity and pyroptosis in HIV-1 infection.

Except for NLRP3 and PYHIN, which were reported more frequently, NLRP1, NLRC4 and NLRC5 inflammasome can also be activated by HIV-1 and are closely associated with HIV-1 infection. Feria et al. found that NLRP1 expression was upregulated in GALT and PBMC of HIV-infected patients compared to controls and that NLRP1 inflammasome activation enhanced HIV-1 replication (Feria et al., 2018). Another study (Dos Reis et al., 2019) has shown that NLRC4 inflammasomes promoted dendritic cell (DC) activation in HIV-1 patients, demonstrating that it plays an essential role in defense against pathogen invasion. Surprisingly, Triantafilou et al. displayed that the HIV-1 envelope protein gp41 can directly activate NLRC4 inflammasome through its cytoplasmic tail (CT), which is the first non-bacterial ligand for NLRC4 reported to date (Triantafilou et al., 2021). Furthermore, an *in vitro* study demonstrated that in murine microglia, regulatory protein Tat of HIV-1 downregulates the production of NLRC5 protein, a negative regulator of the NF-κB signaling pathway, by inducing the upregulation of miRNA-34a, ultimately leading to increased expression of pro-inflammatory cytokines (Periyasamy et al., 2019).

Notably, Pontillo et al. displayed that CARD8 inflammasome plays an important role in HIV-1 infection by SNPs analysis. Among them, the CARD8 rs6509365 polymorphism contributes to co-infection of HIV-1 and Mycobacterium tuberculosis (Pontillo et al., 2013). In addition to gene polymorphisms, Shan et al. recently revealed that CARD8 can act as a sensor of HIV-1 protease activity and initiate inflammasome activation to induce pyroptosis of HIV-1-infected cells (CD4^+^ T cells and macrophages), thereby eliminating latent HIV-1 virus (Wang Q. et al., 2021). Unfortunately, in general, HIV-1 protease activity is slightly active in infected cells prior to viral budding, so it cannot induce CARD8 inflammasomes activation. However, some non-nucleoside reverse transcriptase inhibitors (NNRTIs) induce intracellular HIV-1 protease activity by interacting with HIV-1 Gag-Pol polyprotein and thus are able to clear latent HIV-1 (Kim and Shan, 2022). In addition, some inhibitors of DPPs, such as Val-boroPro and CQ31, have been found to activate CARD8 inflammasome (Rao et al., 2022; Clark et al., 2023). This suggests that targeting the activation of CARD8 inflammasome is an effective approach to clear persistent HIV-1, but how to target specific immune cells (mainly CD4^+^ T cells) to achieve this goal still needs further research.

## 6 Hepatitis B virus and inflammasome

HBV is the typical member of the Hepadnaviridae family, and also is the pathogen that causes Hepatitis B infection. According to the World Health Organization (WHO) reports, about 257 million people worldwide are chronically infected with HBV (World Health Organization, 2017). Although the rate of HBV infection has decreased significantly with the availability of safe and effective preventive vaccine, the burden of disease caused by HBV infection remains a global issue. Notably, chronic hepatitis B (CHB) can lead to further adverse consequences, such as liver cirrhosis and hepatocellular carcinoma (HCC) (Iannacone and Guidotti, 2022). The accumulated data have suggested that in the process of persistent HBV infection, the host innate immunity plays a crucial role (**Table 1**).

**Figure 3:**
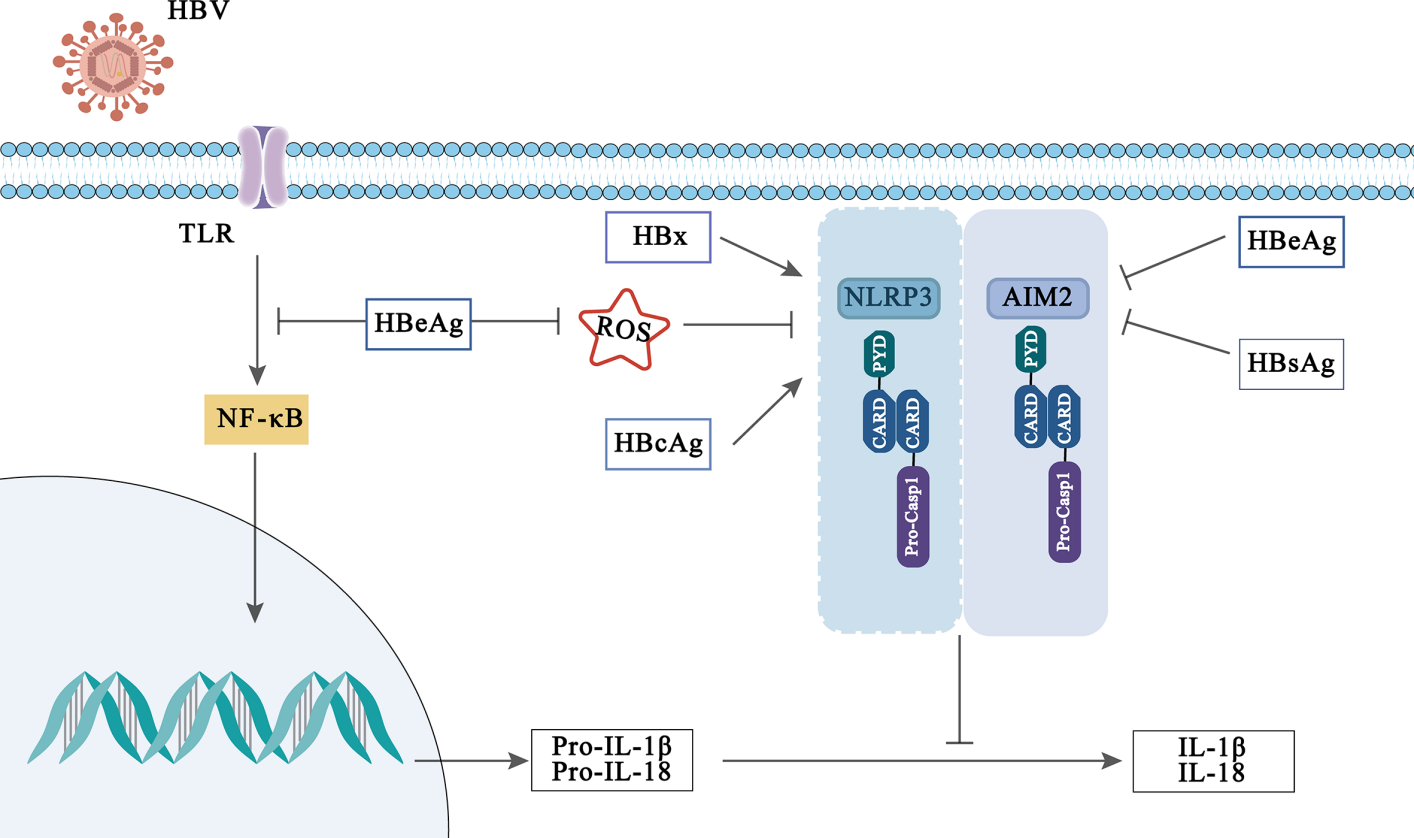
Viral activation and immune evasion of NLRP3 and AIM2 inflammasome by HBV-related proteins.

Inflammasome, as an indispensable component of innate immunity, is vital during HBV infection. First, multiple inflammasomes have been reported to be activated during HBV infection (**Figure 3**). For example, AIM2 and IFI16 of PYHIN family, as dsDNA sensor proteins, can recognize HBV and activate inflammasome (Zannetti et al., 2016; Kumari et al., 2020). In addition, HBV infection may also cause the activation of NLRP3 inflammasome in the liver tissues of patients (Xie et al., 2020). In recent years, an increasing number of studies have reported the role of these inflammasomes in HBV infection. Wu et al. found that AIM2 was expressed in PBMC of patients with HBV infection. Interestingly, AIM2 levels were high in acute hepatitis B (AHB) compared to CHB and negatively correlated with serum HBV viral load and hepatitis B envelope antigen (HBeAg) (Wu et al., 2013), uncovering that AIM2 is involved in HBV immune clearance. However, it has also been shown that AIM2 induces inflammatory damage associated with HBV infection. Han et al. demonstrated that AIM2 expression in hepatocytes was positively correlated with serum HBV viral load. Furthermore, upregulation of AIM2 increases liver inflammation in CHB patients (Han et al., 2015), and this phenomenon has also been observed in patients with HBV-associated glomerulonephritis (Du et al., 2013). The different effects of AIM2 may be related to the cell types in which it is expressed. In addition to AIM2, upregulation of IFI16 mRNA levels was also reported in PBMC of AHB and CHB patients. And, IFI16 expression was negatively correlated with serum HBeAg levels in CHB patients (Chen et al., 2018). Other studies have also confirmed that high expression of IFI16 promotes HBV clearance (Wieland et al., 2004; Yang et al., 2020), implying that IFI16 may be a protective protein during HBV infection. On the other hand, a study disclosed the absence of NLRP1 and NLRC4 activation in the PBMC of CHB patients and it was not associated with HBV-DNA copy and HBeAg status (Askari et al., 2016). These findings lead to a deeper understanding of the inflammasome-mediated innate immunity during HBV infection and provide evidence that these inflammasomes may be potential therapeutic targets for HBV infection.

Interestingly, the influence of inflammasomes in HBV infection may be modulated by HBV-related protein. HBV-related proteins include hepatitis B surface antigen (HBsAg) (which can be divided into small, medium and large sized proteins), hepatitis B core antigen (HBcAg), HBeAg and hepatitis B virus X (HBx) proteins, which played an indispensable role in the pathogenesis and development of HBV infection (Yuen et al., 2018; Xie et al., 2021; Zhao F. et al., 2021). Previously, Lee et al. reported that HBx protein induces IL-18 expression in hepatocytes, but which inflammasome regulates this phenomenon has not been clarified (Lee et al., 2002). While Xie et al. found that HBx protein enhanced NLRP3 inflammasome-mediated inflammation (IL-1β, IL-18 secretion) and cell pyroptosis under oxidative stress in HL7702 cells (Xie et al., 2020). In addition, it is reported that HBcAg can also enhance the activation of NLRP3 inflammasome induced by LPS in HepG2 cells, thus promoting the secretion of inflammatory cytokines (Ding et al., 2019).

However, on the contrary, some evidences have disclosed that HBeAg affects the body’s innate immune response through the inflammasome and participates in the immune escape of HBV infection. A previous report displayed that HBeAg-negative patients can induce PBMC produce higher levels of IL-18, suggesting that HBeAg plays a role in inhibiting IL-18 production (Manigold et al., 2003). Further, Yu et al. indicated that HBeAg inhibits LPS-induced NLRP3 inflammasome activation in Kupffer by inhibiting NF-κB phosphorylation (Yu et al., 2017). In addition, HBeAg has also been reported to inhibit the activation of AIM2 and IFI16 inflammasome in CHB (Chen et al., 2018). Although HBeAg is believed to be a marker of viral replication and to mediate immune escape of HBV infection through inflammasome, other HBV-related proteins or virions also play a key role in maintaining HBV persistence. Whether they can participate in HBV immune escape mediated by inflammasome remains to be further studied and explored.

## 7 Other viruses and inflammasome

In addition to the widely studied viruses mentioned above, other viruses as well as specific proteins from these viruses can also activate different inflammasomes (**Table 2**). For example, Sendai virus (Gram et al., 2012), measles virus (MV) (Komune et al., 2011), HCV (Burdette et al., 2012) and encephalomyocarditis virus (ECMV) viroporin 2B (Ito et al., 2012) have all been verified to activate NLRP3 inflammasome. Meanwhile, dsDNA viruses such as murine cytomegalovirus (MCMV), and vaccinia virus (VV) were recognized by AIM2 (Kumari et al., 2020). Of course, AIM2 has also been demonstrated to play a significant role in RNA virus infection, such as EV71 (Yogarajah et al., 2017) and West Nile virus (WNV) (Ekchariyawat et al., 2015). In addition, IFI16, also a member of the ALR family, was reported to recognize KSHV in endothelial cells (Kerur et al., 2011). Notably, it has been recently reported that viral dsRNA can also induce NLRP6 and NLRP9B inflammasome activation. Among them, the short dsRNA of rotavirus leads to NLRP9B inflammasome activation (Zheng et al., 2020). Interestingly, the activation of these two inflammasomes appeared to contribute to the elimination of the virus. Zhu et al. verified that depletion of intestinal NLRP9B in mice was positively correlated with the susceptibility to rotavirus replication *in vivo* (Zhu et al., 2017), and NLRP6 may play a vital role in enteric ECMV clearance (Wang P. et al., 2015). Similarly, NLRP3 appears to play a protective role in WNV and ECMV infections (Kanda et al., 2000; Ramos et al., 2012). However, there are also some studies have shown that NLRP3 seems to induce inflammatory response during viral infection. For example, HCV triggers liver inflammation via NLRP3 inflammasome (Farag et al., 2017). These suggest that NLRP3 may play different roles in different viral infections in different cells, and further discussion is needed.

**Table 2:** Different viruses and viral proteins participate in the activation of inflammasomes and the immune escape mediated by inflammasomes.

Inflammasome	Cells expressed	Viral/viral proteins that activate the inflammasome	Viruses/viral proteins involved in immune escape	References
NLRP1	human keratinocytes and PBMCs	3C protease of human rhinovirus	Bovine pox virus F1L protein	(Gerlic et al., 2013; Mitchell et al., 2019; Robinson et al., 2020)
		3C protease of coxsackievirus B3	KSHV ORF63 protein	(Gregory et al., 2011; Robinson et al., 2020)
		KSHV ORF45 protein		(Yang et al., 2022)
		Coronavirus 3CL protease NSP5		(Planes et al., 2022)
		HIV-1		(Feria et al., 2018)
NLRP3	Immune cells of the myeloid lineage like neutrophils, monocytes, and dendritic cells, barrier cells, lymphocytes and neurons	E, N and ORF3a proteins of SARS-CoV-2	NSP1 and NSP13	(Siu et al., 2019; Kim et al., 2021; Pan et al., 2021; Zhang Y. et al., 2021; Zheng M. et al., 2021; Sun et al., 2022; Xu et al., 2022)
		IAV	MV V protein	(Komune et al., 2011; Kuriakose et al., 2016; Gaba et al., 2019)
		HBx proteins and HBcAg	HBeAg	(Yu et al., 2017; Ding et al., 2019; Xie et al., 2020)
		HIV-1 regulatory protein Tat, accessory protein Vpu and glycoprotein gp120	Sendai virus V protein	(Rossol et al., 2012; Komatsu et al., 2018; He et al., 2020; Triantafilou et al., 2021)
		Sendai virus	KSHV Orf63 protein	(Gram et al., 2012)
		MV	EV71	(Komune et al., 2011; Wang H. et al., 2015)
		HCV		(Burdette et al., 2012)
		ECMV viroporin 2B		(Ito et al., 2012)
NLRC4	Myeloid cells	HIV-1 envelope protein gp41		(Sutterwala and Flavell, 2009; Triantafilou et al., 2021)
AIM2	Lymph nodes, appendix and spleen	Human cytomegalovirus	HBeAg	(Huang et al., 2017; Chen et al., 2018)
		Human papilloma virus	poxviruses	(Reinholz et al., 2013; Khare et al., 2014)
		Hepatitis B virus		(Pan et al., 2016)
		SARS-CoV-2		(Junqueira et al., 2022)
		IAV		(Zhang et al., 2017)
		HBV		(Wu et al., 2013)
		HIV-1		(Burckstummer et al., 2009; Fernandes-Alnemri et al., 2009)
		MCMV, VV and EV71		(Yogarajah et al., 2017)
		WNV		(Ekchariyawat et al., 2015)
IFI16	Vascular endothelial cells, keratinocyte cells and hematopoietic cells	EBV	HPV	(Ansari et al., 2013; Caneparo et al., 2018; Song et al., 2020)
		HSV	HBeAg	(Johnson et al., 2013; Chen et al., 2018)
		HBV		(Chen et al., 2018)
		HIV		(Nissen et al., 2014)
		KSHV		(Kerur et al., 2011)

However, not surprisingly, some viruses have also evolved different strategies to inhibit the assembly and activation of inflammasome in order to evade host immunity during infection and promote persistent viral replication. For example, the MV V protein (Komune et al., 2011), Sendai virus V protein (Komatsu et al., 2018) and KSHV ORF63 protein can inhibit NLRP3 assembly by interacting with it. At the same time, the KSHV ORF63 protein was found to be a homolog of NLRP1, so it can block the activation of NLRP1 inflammasome (Gregory et al., 2011). In addition, some poxviruses express POPs (a class of proteins containing only the PYD), which inhibit the activation of caspase-1 by competing with ASC for the PYD domain in AIM2, thereby preventing the assembly of AIM2 inflammasome (Khare et al., 2014). Moreover, it also restrains caspase-1 activation through serpin, a crmA (SPI-2) gene coding inhibitor (Kettle et al., 1995). On the other hand, certain viruses promote the degradation of inflammasome. For instance, EV71 induces NLRP3 degradation by cleavage of viral proteases 2A and 3C (Wang H. et al., 2015). Finally, even after the successful activation of inflammasomes, viruses may still prevent the effector function of cytokines, such as IL-1βand IL-18, produced by them. Like VVs, they can encode IL-1β-binding proteins (IL-1β BP) and IL-18-binding proteins (IL-18BPs) (Alcamí and Smith, 1992).

## 8 Clinical therapies targeting the inflammasome

As described above, activation of inflammasome is involved in the pathogenesis of different viral infections, which has given rise to widespread interest in inflammasome inhibitors for the treatment of viral infections. Some medications targeting inflammasome have been clinically studied in patients and shown to potentially improve survival rate or survival time. In addition, some small molecule compounds that directly/indirectly target inflammasome activation or caspase have been verified to effectively block inflammatory effects in cellular or animal experiments, but further researches are needed to validate.

To date, several NLRP3 protein inhibitors have been reported to restrain inflammatory effects in viral infection. For example, MCC950 is a direct inhibitor of NLRP3 protein. It was demonstrated in juvenile mice that after administration of MCC950 in 3 days after IAV infection, the activation of NLRP3 inflammasome was inhibited, which reduced IL-18 secretion into alveolar space and improved the survival of these mice (Lučiūnaitė et al., 2022). Moreover, it was able to reduce lung injury in IAV-induced COPD rats, thereby improving survival time (Ji et al., 2022). Besides, OLT1177 (Dapansutrile) is a kind of β-sulfonyl nitril compound and is also the only specific NLRP3 inhibitor with ora activity. Its safety and efficacy was evaluated by a randomized controlled trial in moderate COVID-19 (NCT04540120) (Bonaventura et al., 2022). Another specific NLRP3 inhibitor, tranilast, is attached to the NACHT domain to prevent NLRP3-NLRP3 contact and consequent oligomerization, but not effect on its ATPase function, thereby potentially reducing pyroptosis (Saeedi-Boroujeni et al., 2021).A randomized controlled trial conducted in non-ICU-admitted hospitalized patients with COVID-19 found that, compared with control group received antiviral treatment, patients treated with tranilast had lower levels of Neutrophil-to-Lymphocyte Ratio (NLR), q- C-reactive protein (q-CRP), IL-1, TNFα and lactate dehydrogenase (LDH) (Saeedi-Boroujeni et al., 2022). Moreover, Colchicine is an anti-inflammatory medication with the ability to inhibit the oligomerization of NLRP3 inflammasome, which may limit the release of IL-1 and block the excessive injury of downstream cytokines, such as IL-6 and TNFα (Bonaventura et al., 2022). There is a study that patients with COVID-19 treated with colchicine had a higher survival rate than the standard-of-care group at 21 days of follow-up (Scarsi et al., 2020). However, in another randomized, controlled, open-label, platform trial, colchicine did not reduce COVID-19 adult inpatients’ 28-day mortality, duration of hospital stay, or risk of progressing to invasive mechanical ventilation or death (Group, 2021). This may indicate the limited utility of colchicine in COVID-19. Berberine, an isoquinoline alkaloid extracted from several commonly used Chinese herbs, further limited NLRP3 activation by inducing mitochondrial autophagy, and thus reducing ROS production. Berberine attenuated lung injury in IAV-infected mice and reduced mortality in influenza viruses pneumonia (Liu et al., 2020). Unfortunately, inhibitors targeting the NLRP3 inflammasome appear to play a therapeutic role on the basis of inflammatory cytokine storms. So there doesn’t seem to be much research into some viral infections that don’t produce inflammatory cytokine storms. However, interestingly, inhibition of DPP9, an negtive regulator of CARD8 inflammasome, was able to kill HIV-1-infected cells in the absence of NNRTIS and promotes the clearance of HIV-1 infected cell both *in vitro* and in humanized mice by collaborating with NNRTIS (Clark et al., 2023). This provides a strong basis for future treatment of inflammasome in different viruses. In addition, although there are other inflammasome known to be associated with viral infections, such as NLRP1 and AIM2, and no specific inhibitors have been identified to directly target these inflammasome to regulate inflammation.Inhibitors of caspase have mostly been studied in animal or cellular experiments, but unfortunately no clinical progress has been achieved. The caspase-1 inhibitor AC-YVAD-CMK or PAN-caspase inhibitor Z-VAD-FMK reduced IL-1β produced by SARS-CoV-2 infected monocytes. Meanwhile, they suppressed the replication of IVA, resulting in a decrease pro-inflammatory cytokines and chemokines (such as IL-9, IL-6, TNF-α and MCP-18) (Liu et al., 2014). PAN-caspase inhibitor emricasan inhibits caspase-1 activity of CD4^+^ T lymphocytes in the peripheral blood of patients with moderate to severe COVID-19 (Plassmeyer et al., 2022). Glyburide, a sulfonylurea drug for diabetes is a caspase-1 inhibitor, saliently restricted bovine herpesvirus 1 (BoHV-1) infection (Wang et al., 2014) and reversed SARS-CoV-2-induced cell death (Cheon and Koo, 2021).

Recently, greater progress has been made in studies related to inhibitors of downstream cytokines of inflammasome. Previously, the recombinant IL-1R antagonist anakinra was approved for the treatment of rheumatoid arthritis, refractory gout and chronic auto-inflammatory disorders. Recently, the U.S. Food and Drug Administration (FDA) has issued an Emergency Use Authorization (EUA) for the emergency use of anakinra to treat COVID-19 in hospitalized adults with positive results of direct SARS-CoV-2 viral testing with pneumonia requiring supplemental oxygen. However, the phase 1 clinical trial for anakinra treating neuroinflammation of patients with HIV-1 infection has been terminated for various reasons (NCT02527460).

However, it is worth noting that most of these inhibitors are non-specific, have low efficacy, and often affect other inflammatory pathways. Moreover, the potential risk of cross-reactivity is high. In addition, inhibitors targeting IL-1β or IL-18 can lead to unintentional immunosuppressive effects. Therefore, inhibitors targeting inflammasome pathways have not been currently widely used in viral infectious diseases. This further raises expectations about how new drugs targeting the inflammasome pathway will achieve higher efficacy and fewer adverse effects.

## 9 Conclusion and perspectives

In the immune response of virus infection, the activation of inflammasome is a key aspect. In recent years, with the development of research on inflammasome, mechanisms of some inflammasome assembly and activation have been gradually clarified. At present, it has been proved that inflammasome are activated by many viruses. However, it remains to be seen how the ALR/NLR proteins accurately sense the virus, how the different inflammasome complexes interact with each other, and how the inflammasomes interact with other PRRs to successfully respond to viral infections.

Not only that, although current research uncovers that inflammasome and its mediated pyroptosis play an important role in viral infection, more data are needed to support the benefits to the host. On the one hand, current studies suggest that inflammasome activation and inflammatory factors inducted by them can eradicate virus by eliminating virus-infected cells in the body through pyroptosis. Some viruses express molecules that inhibit the assembly and activation of inflammasomes, which can also be confirmed from the side. On the other hand, however, abnormal regulation of inflammasome signaling and the associated abnormal secretion of pro-inflammatory cytokines contribute to the occurrence of chronic inflammation, thereby driving viral replication. These phenomena unravel an intriguing and challenging complex mechanism of the inflammasome in viral infection. Not surprisingly, different inflammasomes may play different roles, but it is worth noting that the same inflammasome may have different effects on the body, depending on cell type and infection stage. For example, NLRP3 may play an anti-infective role in the early stage of viral infection, but the abnormal regulation of its signaling will lead to chronic inflammation, thus driving viral replication. We also summarized these differences (**Table 1**). But the mechanism still needs to be further explored.

Not surprisingly, the immune escape of viral proteins against inflammasome, however, unfortunately, the factors reported so far have not yet played a decisive role in the suppression or escape of the virus, which may be related to the fact that the human body itself is a complex whole. But, great progress has been made in the development of inflammasome activators/inhibitors, which provides new ideas for the development of new therapeutic drugs and vaccines. Given the diversity of pathogen and host-derived factors and their complex interactions, targeting inflammasome remains a challenge. Further research is needed to determine the clinical therapeutic potential of targeting inflammasome complexes during viral infection.

Collectively, understanding the complex interplay between viral infection and inflammasome activation will improve the understanding of the pathological mechanisms of corresponding diseases and provide better treatment strategies. Therefore, it is urgent to reveal the role and mechanism of inflammasome activation in viral infection.

## Author contributions

Conception and design: HB and JQ. Collection and assembly of data: NW, CZ, JX, SM, HJ, MY. Data analysis and interpretation: NW, CZ, FA. Manuscript writing: NW, CZ, HB and JQ. Administrative support: YZ and HB. Final approval of manuscript: All authors. All authors contributed to the article and approved the submitted version.

## Glossary

**Table:**

AIM2	absent in melanoma 2
AIDS	acquired immunodeficiency syndromes
AHB	acute hepatitis B
ALRs	AIM2-like receptor
BIR	Baculovirus inhibitor-of-Apoptosis repeat
BoHV-1	bovine herpesvirus 1
CARD	caspase activation and recruitment domain
CLRs	C-type lectin receptors
COVID-19	Coronavirus Disease, 2019
COUP-TF1	chicken ovalbumin upstream promoter transcription factor 1
CT	cytoplasmic tail
CHB	chronic hepatitis B
cGAS	cyclic GMP-AMP synthase
DAMPs	damage-associated molecular patterns
DC	dendritic cell
DPP	dipeptidyl peptidases
dsDNA	double-stranded DNA
EBV	epstein-Barr Virus
ECMV	encephalomyocarditis virus
EUA	Emergency Use Authorization
EV71	enterovirus 71
EGCG	epigallocatechin gallate
FDA	Food and Drug Administration
FIIND	function-to-find domain
GALT	gut-associated lymphoid tissue
GSDMD	gasdermin D
HBV	hepatitis B virus
HCC	hepatocellular carcinoma
HD	Helical Domain
HIV-1	human immunodeficiency virus type 1
HPV	Human papillomavirus
HCV	hepatitis C virus
HSV-1	herpes simplex virus 1
HBsAg	hepatitis B surface antigen
HBcAg	hepatitis B core antigen
HBeAg	hepatitis B envelope antigen
HBx	hepatitis B virus X
HMGB-1	high mobility group box 1
IAV	Influenza A viruses
IFI16	interferon-inducible protein 16
IFN-I	type I IFN
KSHV	Kaposi sarcoma-associated herpesvirus
LF	lethal factor
LeTx	lethal toxin
LPS	lipopolysaccharide
LRRs	leucine-rich repeats
MAVS	mitochondrial antiviral signaling protein
MxA	myxovirus resistance protein 1
MYD88	myeloid differentiation factor 88
MCMV	murine cytomegalovirus
MV	measles virus
NBD	nucleotide-binding domain
NOD	nucleotide-binding oligomerization domain
NNRTIs	non-nucleoside reverse transcriptase inhibitors
NSPs	non-structural proteins
NEK7	NIMA-related kinase 7
NLRs	NOD-like receptors
NAIP	neuronal apoptosis inhibitory protein
ORF	open reading frame
PB1	polymerase basic protein 1
PRRs	pattern recognition receptors
PAMPs	pathogen-associated molecular patterns
PYD	pyrin domain
RIG	retinoic acid-induced gene
RXR-α	retinoid X receptor-α
ROS	reactive oxygen species
RLRs	RIG-I-like receptors
SARS-CoV-2	severe acute respiratory syndrome coronavirus 2
SNPs	single nucleotide polymorphisms
SIV	simian immunodeficiency virus
STING	stimulator of interferon genes
TLRs	toll-like receptors
TRIM	Tripartite motif-containing protein
VV	vaccinia virus
VSV	vesicular stomatitis virus
WNV	West Nile virus
WHO	World Health Organization
WHD	Winged Helix Domain
ZBP1	Z-DNA binding protein 1
3’-UTR	3’-untranslated region.
